# HBZ inhibits the HAT activity of the cellular coactivators p300 and CBP

**DOI:** 10.1186/1742-4690-8-S1-A150

**Published:** 2011-06-06

**Authors:** Diana Wright, Torsten Wurm, Nicholas Polakowski, Jean-Michel Mesnard, Isabelle Lemasson

**Affiliations:** 1 Dpt. Microbiology and Immunology, East Carolina University, Brody School of Medicine, Greenville, NC, 27834, USA; 2 Centre d’études d’agents Pathogènes et Biotechnologies pour la Santé, CNRS/UMR 5236/CNRS, UM1/UM2, Montpellier, 34923, France

## 

HBZ is one of the HTLV-1-encoded proteins known to affect transcription. We previously found that this viral protein interacts with the homologous cellular coactivators p300 and CBP. These large coactivators play an integral role in regulating expression of numerous genes, as they are known to bind more than 400 proteins that function to regulate transcription. They also contain histone acetyltransferase (HAT) activity that prominently targets specific lysine residues in the histone proteins of chromatin. Acetylation of histones is a hallmark of active transcription. In addition, p300 and CBP are also able to acetylate specific transcription factors, affecting the cellular localization and/or transcriptional activity of these factors. The HAT domain was one of coactivator domains we found to be directly contacted by HBZ. Using in vitro HAT assays with recombinant proteins, we have determined that HBZ strongly inhibits the HAT activity of these coactivators. This inhibition not only affects histone acetylation but also the acetylation of transcription factors, such as p53 and p65. In the case of these factors, a decrease in acetylation is known to reduce their activity. Interestingly, we found that inhibition of HAT activity is mediated through the bZIP domain of HBZ rather than the activation domain. Significantly, we confirmed these effects on histone, p53 and p65 acetylation in HeLa and Jurkat cells expressing HBZ. Given the fact that the HAT domain in p300/CBP is mutated or deleted in certain cancers, HBZ inhibition of the HAT activity may be important for progression of ATL.

